# The relationship between capacity and credibility: implications for epistemic injustice

**DOI:** 10.1093/medlaw/fwaf039

**Published:** 2025-11-04

**Authors:** Ruby Reed-Berendt, Agomoni Ganguli-Mitra

**Affiliations:** School of Law, University of Edinburgh, Old College, South Bridge, Edinburgh EH8 9YL, United Kingdom; School of Law, University of Edinburgh, Old College, South Bridge, Edinburgh EH8 9YL, United Kingdom

**Keywords:** best interests, credibility, epistemic exclusion, epistemic injustice, mental capacity, mental disability

## Abstract

In this article, we analyse the concept of mental capacity through considerations of epistemic injustice. We suggest that an assessment of a person’s capacity will always involve consideration of their epistemic credibility. Understanding capacity assessments in this manner allows us to illustrate the epistemic exclusions and injustices that can arise. First, attitudes and stereotypes about mental disability and illness, as well as characteristics such as gender and race, can make a significant difference in terms of who is believed and considered credible. We raise concerns for the potential of these biases to influence capacity assessments inadvertently. Secondly, a person deemed to lack capacity has their epistemic agency significantly curtailed; their contributions to the decision-making process are not those of a full epistemic agent, but those of a derivatized subject, giving rise to epistemic exclusion. We further argue that capacity determinations rely on specific hermeneutical resources, namely those based in medical-scientific or legal knowledge, which may be inapt for interpreting the experiences of disabled people and those with mental illness. Finally, we highlight important insights that this approach provides for considering options for reform in practice and in law.

## I. INTRODUCTION

Questions of epistemic injustice have become an important concern for health ethics and disability scholarship across a variety of contexts.^1^ An analysis of epistemic injustice directs our attention towards forms of injustice that arise from processes of knowledge generation and exchange; it entails consideration of how epistemic practices and structures unjustly disregard or distort certain forms of knowledge.^2^ An important concern of epistemic injustice is the ways in which certain people, due to structures of power and relations of domination and oppression, are not considered legitimate or credible epistemic agents, that is, ‘agents who are able to utilize persuasively shared epistemic resources within a given community of knowers in order to participate in knowledge production’.^3^ Instead, they are unjustly silenced or denied opportunities to contribute to epistemic systems and processes.^4^ Applying such concerns to mental illness and disability, various commentators have pointed to how psychiatric practices and mental health legislation can produce epistemic injustices—particularly by denying the epistemic agency of mentally disabled people.^5^ However, limited work has been done to date on the relevance of these concerns to the concept of mental capacity and how forms of injustice arise through capacity-based legislation.^6^ Mental capacity is used across a variety of jurisdictions to assess whether a person is considered capable of making a decision for themselves. Persons who are deemed to lack capacity are not legally considered able to make decisions; instead, they are made subject to substitute decision-making regimes, where decisions about their lives are made by another person (frequently an appointed guardian, health and social care professional, or a court).

In this article, we analyse the concept of mental capacity through the lens of epistemic injustice. Focusing on mental capacity as defined under the Mental Capacity Act 2005 (MCA) in England and Wales, we suggest that assessing a person’s capacity also involves considerations of the person’s credibility; the assessor will also evaluate whether a person can be considered relevantly authoritative when accounting for their decision-making process and whether they are able to give a convincing and true account of their views and experiences. Recognizing this, we highlight three ways in which the assessment of capacity can give rise to instances of injustice that are specifically epistemic, including forms of testimonial and hermeneutical injustice. This can provide important insights into how aspects of legislative frameworks, such as the MCA, and their application in practice, can be problematic. We then highlight the potential this offers when considering possible reforms in law and practice. At a time where debates are ongoing regarding the future of capacity law, and the extent to which the concept should be retained in light of critiques based on the UN Convention on the Rights of Persons with Disabilities (CRPD),^7^ an epistemic injustice lens provides a useful critical tool to consider not only the pitfalls of existing legislative frameworks and processes, but also the desirability of different kinds of reform.

## II. FORMS OF EPISTEMIC EXCLUSION AND INJUSTICE

Consideration of epistemic questions is a key marker of feminist approaches to health and health ethics. Feminist standpoints bring attention to the ways in which knowledge about health and the self are uniquely informed by an individual’s social position. Given the nature of existing structures, processes, and power, an individual’s epistemic authority and contribution can also be questioned, thwarted, or entirely ignored within healthcare and beyond, often along the lines of gender, race, disability, class, and other intersecting identities. This gives rise to a specific type of wrong, which has come to be referred to as epistemic injustice: ‘forms of unfair treatment that related to issues of knowledge, understanding, and participation in communicative practices’.^8^

The literature on epistemic injustice elucidates many varieties and forms of injustices, and there is no space here to cover them all.^9^ The concept is frequently associated with Miranda Fricker, and indeed her work and coinage have been generative for scholarship in the area.^10^ However, analysing epistemic practices and structures has long been a key part of Black feminist, critical race, and decolonial scholarship, which has called attention to *inter alia* the ways in which Black women’s knowledge is undervalued within academic disciplines and activism, and the routine silencing and discrediting of Black and colonized populations and the knowledges they produce.^11^ This speaks to the importance of not foreclosing on other ways of knowing by defining epistemic injustice overly narrowly.^12^

For the purposes of our analysis, *epistemic agency* refers to an agent’s ability “to interact with other members of their epistemic community”—to participate in processes of knowledge generation and use shared epistemic resources; to be recognized and acknowledged by others as a credible knower.^13^  *Epistemic exclusion* takes place where a person’s epistemic agency is compromised, for example, if they are unable to use or access epistemic resources or contribute to their production.^14^ As Scully highlights, such exclusion ‘frequently (perhaps inevitably) follows from social and political oppression driven by existing and historical power relations’^15^ and can be contrasted with *epistemic privilege*, where the knowledge of dominant groups is preferentially absorbed into shared epistemic resources.^16^  *Epistemic injustice*, then, refers to forms of injustice that result from epistemic exclusion of marginalized social groups and how such persons have their epistemic agency unjustly infringed upon.^17^ We highlight key instances of injustice that are relevant to our discussion.

*Testimonial injustice* occurs during speech acts, where a hearer fails to give credibility to the words of a speaker due to some form of prejudicial identity stereotype.^18^ This failure to accord credibility harms the speaker’s epistemic agency; the hearer does not acknowledge them as a credible epistemic agent who is to be believed and recognized as authoritative.^19^ As such, credibility in this context is not only dependent on the ability of an epistemic agent to signal that they have knowledge and authority; it is also dependent on the hearer’s ability to recognize and accept such authority and view them as a legitimate possessor of knowledge.^20^ Importantly, attributions of deflated credibility to an epistemic agent result in a harm that goes beyond the immediate action^21^—it can significantly curtail the scope of their interaction with others, or exclude them from their relevant epistemic community, because they are no longer trusted as a credible agent.^22^ Testimonial injustice is thus primarily concerned with faulty or unwarranted assessments of a person’s credibility during exchanges between a speaker and a hearer. Injustices can also arise through shortcomings in how credibility is attributed to particular social groups, creating unfair disparities in credibility and authority between members of social groups (often in a manner corresponding with existing patterns of domination and oppression) and leading to the ‘privileged epistemic treatment of some […] and the underprivileged epistemic treatment of others’.^23^

*Hermeneutical injustice* arises not in testimony but out of insufficiencies or gaps in shared epistemic resources,^24^ where epistemic systems are skewed and biased towards the accounts of dominant groups and can instead be inapt for (or incapable of) understanding the social experiences of marginalized groups.^25^ The key problem of concern here is not questions of credibility but structurally prejudiced epistemic structures and systems, and the ways in which these unjustly distort or hide certain kinds of experiences. As we will argue below, the hearer’s (and at times the broader system’s) ability to confer due credibility is heavily reliant on the tools available to both hearer (and the system) and the speaker (shared interpretations of what constitutes harm or suffering, for example, and socially accepted standards around language). In the context of health care, Carel and Kidd highlight that:

even if the patient’s testimony were relevant, emotionally balanced and so on, what they say is not expressed in the accepted language of medical discourse and will therefore be assigned a deflated epistemic status.^26^

*Contributory injustice* sits between agential and structural forms of epistemic injustice, occurring where a hearer could make use of alternative epistemic resources to understand and recognize the speaker’s testimony, but wilfully continues to rely on dominant (and structurally prejudiced) epistemic resources.^27^ Through this, the speaker’s ability to exert epistemic authority and contribute to shared epistemic resources is curtailed.^28^ Contribution to shared knowledge and knowledge-making is therefore only possible when an agent’s testimony is recognized as credible and authoritative, *and* where there is a willingness to modify existing tools and standards and overcome structurally entrenched prejudices. As Carel and Gyöffry suggest, ‘denying someone credibility they deserve is one form of epistemic injustice; denying them the role of a contributing epistemic agent at all is a distinct form of epistemic exclusion.’ ^29^

When considering how such concerns of injustice can apply to capacity, there are two important elements of context to remember. First, as Scrutton has argued, a focus on epistemic injustice in mental health (including, we would argue, mental capacity) cannot ignore the ontological status of mental illness—and how epistemic injustice can arise from the categorization of experiences and their interpretation in ‘purely medical terms’, rather than recognizing the role of social structures and causes.^30^ In other words, because mental illness and disability are so closely associated with the ability to make use of epistemic tools, speakers with such diagnoses will by default be at a disadvantage in terms of their credibility and authority. As Leblanc and Kinsella argue, ‘negative perceptions of Mad persons as delusional, emotionally unstable, unpredictable, untruthful, untrustworthy, and lacking all capacity for “rational thought” often lead to their being discredited as legitimate knowers’.^31^ Moreover, it is a well-documented phenomenon that patients are deemed more credible and authoritative when they are able to use terms, phrases, and expressions that match the expectations of the medical or legal professional.^32^ As such, it is important to focus not just on issues of testimonial failure but also on how the epistemic tools, resources, cultures, and practices of the medico-legal landscape are influenced by dominant groups and the ways in which they can fail to account for certain experiences.

Second, as the literature makes clear, law and legal processes can give rise to epistemic injustices. For example, disability scholars such as Campbell suggest that negative perceptions of disability are built into legal frameworks, and these feed into prejudicial stereotypes that negatively influence the credibility afforded to disabled people.^33^ Moreover, court proceedings (especially in the criminal context) can be liable to produce testimonial injustice, through failures of juries or legal professionals to accord credibility to witnesses or complainants due to prejudice.^34^ Bringing these two contexts together, Newton-Howes and colleagues suggest that the existence of mental health law can lead to legally sanctioned forms of epistemic injustice, by legalizing ‘the idea that people with mental illness are epistemically of less account than others’ and altering expectations for how doctors ought to act regarding the knowledge of their patients.^35^ As we argue below, this same issue—of legally sanctioned injustices—should also be a pertinent concern for mental capacity legislation and how it is applied, both in terms of day-to-day decisions, and in court spaces (although we recognize the issues arising are potentially different). Because (as we suggest) an assessment of capacity entails judgments on credibility, it is particularly prone to allowing testimonial, hermeneutical and contributory limitations, and injustices to flourish. We now turn to consider this.

## III. MENTAL CAPACITY AND CREDIBILITY

Approaches to mental capacity vary by jurisdiction, but they can generally be defined as status-based (where lack of capacity is determined by reference to a specific characteristic, such as age or disability), functional-based (which considers a person’s cognitive abilities in decision-making), or a combination of the two.^36^ Functional tests are often said to be less discriminatory, in that they do not entail a global denial of a person’s capacity and legal agency based on the mere existence of disability, but instead require a specific assessment of a person’s ability to make particular decisions at a given moment in time.^37^ Such an approach is taken under the MCA in England and Wales, which we focus on for the purposes of this article; however, we consider that our arguments have resonance for other jurisdictions that also utilize a functional or combined approach to capacity.

Under the MCA, a person is deemed to lack capacity if they are assessed as unable to make a specific decision as a result of mental disability or cognitive impairment.^38^ This assessment is generally undertaken by a health and social care professional,^39^ although legal professionals (including judges) may also carry this out.^40^ A person is considered ‘unable to make a decision’ if they are unable to understand, use, retain, and/or weigh the information that is relevant to that decision, or communicate that decision.^41^ This is exemplary of the functional approach to capacity, albeit the MCA (and many similar legislative instruments) only applies in situations where a person’s mental disability or impairment is *causative* of their inability to make decisions.^42^ As such, we cannot lose sight of the reality that only persons with a diagnosed or suspected mental/cognitive impairment will have their capacity called into question (and the relevance of the ontological status of mental illness noted above).

The MCA can be used to assess decision-making ability across a wide range of contexts. A person’s capacity can be assessed in relation to a decision to consent to or refuse a particular medical treatment, deciding where they live and what care they receive, a decision to enter a contract or to undertake litigation, or even decisions to form sexual and intimate relationships with others. A person deemed to lack capacity is not considered legally capable of making the particular decision for themselves; instead, it is made based on an assessment of their ‘best interests’.^43^

Considering this process through the lens of epistemic injustice, the assessment of capacity under the MCA, especially in health and social care settings, is recognizably a form of testimonial exchange between that person and the assessor (who will usually be a health and social care professional). It is undertaken via speech acts between the two parties, with a heavy focus on the person’s testimony (ie the person acts as the ‘speaker’ noted in Section II above, and the assessor as the ‘hearer’). The process requires the assessor to provide the person with information (with visual or auditory aids as required), ask questions, and take account of their responses, before deciding whether the person demonstrates that they understand this information, can remember it, and are able to make use of it in the decision-making process.^44^ As Fricker suggests, in any face-to-face testimonial exchanges, there *must* be some attribution of credibility (or otherwise) by the hearer regarding the speaker.^45^ Therefore, during any assessment of capacity, the assessor/hearer will inexorably have to decide whether they attribute credibility to the person/speaker’s word. Otherwise stated, whether a person is deemed to have capacity will depend on whether they, as a speaker, have been able to present themselves as credible and intelligible in ways that are considered appropriate.

An illustration of this can be found in the case of *AH.*^46^ This case concerned ‘Anna’, a 46-year-old woman diagnosed with mild learning disability and suspected personality disorder, who also had diabetes. She was assessed as lacking capacity by the consultant psychiatrist in relation to decisions around residence and care for diabetes. Anna had been discharged to a care home following a stay in hospital because of complications from diabetes. She wished to return home and had developed her own care plan detailing the support she wanted. She was deemed to lack capacity because she lacked an understanding of her needs and was incapable of using/weighing information due to her ‘extreme egocentricity and rigidity and refusal to take reality or other views into account’.^47^ In particular, the psychiatrist noted that the care plan that Anna wanted to be put in place was not possible, but she was unable to recognize this because, due to her learning disability and personality disorder, she did not accept that she had mental health needs.^48^ Here, we would argue, the consultant psychiatrist also did not consider Anna to be a credible epistemic agent; because her views did not reflect ‘reality’, because she was abnormally rigid and did not listen to others (as a result of her diagnosis)—her testimony and knowledge (ie the care plan she created and her expressed desire for this to be followed) were not considered credible.

Beyond this basic premise, we would argue that during a capacity assessment, the assessor/hearer is actively encouraged to consider (and potentially question) whether the person/speaker is a credible epistemic agent. Elements of the assessment can include whether a person can recall and repeat information back to the assessor, whether they use information in the appropriate manner, if they can make choices and offer reasons for those choices, reflect on the issues, and be settled in their views.^49^ These kinds of attributes speak closely to whether a person’s views appears trustworthy or believable—that is, whether they are credible. As Medina suggests, judgments around another person’s credibility are not divorced from social positionality and ideas of normalcy; rather, hearers will be considering whether a speaker is credible by reference to their ideas of what a trustworthy and credible agent looks like.^50^ Capacity assessments operate in just this manner; they involve contrasting the cognitive decision-making processes of the person being assessed with a baseline of ‘normal’ decision-making (ie using information in the right way, offering reasons for choices, showing the ability to reflect, etc.).^51^ Note how this happened in the *AH* case noted above—the attributes emphasized about Anna (her rigidity and failure to listen) highlighted the ways in which her decision-making and testimony failed to demonstrate appropriate cognitive processing. This contrastive process not only opens the door to a greater consideration of credibility, but the act of assessment also involves interrogating the person’s knowledge and whether their views are trustworthy, placing them in a position where their credibility as an epistemic agent is already in question.

Another important feature to recognize is that how credibility is conferred also depends on the epistemic tools (including words, body language, phrases, connections, etc) that are available to and used by the parties in the testimonial exchange. As the epistemic injustice literature recognizes, not all words or forms of expression are considered credible by hearers. For example, writing in the context of the doctor–patient relationship, Carel and Kidd have demonstrated how medically trained professionals are constantly evaluating which aspects of communication and information are relevant to their assessment:

A common complaint from clinicians is that patients’ speech is full of irrelevant information, that patients are (understandably) upset and therefore can be irrational, and that listening for medically relevant information precludes listening to other information conveyed in patient speech (such as existential concerns, need for empathy, or emotional content).^52^

The MCA mandates a very specific set of epistemic tools that form the basis of capacity assessments—including, crucially, the identification and exchange of information deemed relevant to the particular ‘matter’.^53^ As recent case law has emphasized, it is for the assessor (either the professional or the Court) to identify and determine what the ‘matter’ in question is, and therefore what information is relevant to that decision.^54^ As such, the information that is exchanged, and the precise ‘matter’ that is being assessed, is determined not by the speaker, but by the hearer. As we will argue below, this can see certain elements of a person’s testimony disregarded as irrelevant because they do not fit with the epistemic tools made available by the MCA.

Considering the above, we would suggest that a person will usually be deemed to have capacity in relation to this identified matter if the hearer recognizes them as epistemically authoritative—they need to offer a credible account of their decision-making and demonstrate they can act as a trustworthy epistemic agent.^55^ Consequently, a person who is considered to lack capacity is, to at least some extent, subject to epistemic exclusion, in that they are no longer acknowledged (in and by law) as a credible knower who is able to make a decision based on their own narrative or judgments. Instead, they are subject to best-interests decision-making, where another person determines the appropriate course of action (we discuss the implications of this further below).^56^ In this manner, their epistemic agency is necessarily curtailed—there is a restriction on the ‘scope and type of their interactions with other epistemic agents’,^57^ which now take place through the prism of the best interests process.

Reflecting on this, like mental health law, capacity law can also be described as operating to delineate between persons who are considered reliable and credible epistemic agents, and those whose epistemic agency is legally questionable.^58^ And as the implications of being considered to lack credibility are significant, and it is worth being attentive to situations where attributions of credibility may be problematic (eg unwarranted, or faulty in the direction of excess or deficit) and therefore issues of epistemic injustice.

## IV. CONCERNS OF EPISTEMIC INJUSTICE

Not all instances of deflated credibility—or indeed epistemic exclusion—can be considered an injustice *per se*. As Scully argues, there can be good reasons why some kinds of knowledge ought to be excluded from shared resources, such as where knowledge is harmful or where, upon consideration, it appears to be truly irrelevant.^59^ Fricker similarly acknowledges that deflated credibility is not an injustice on its own, focusing on forms of discrimination and prejudice that render assessments of credibility faulty.^60^ However, recognizing that conferrals of credibility take place when capacity is assessed and that this can facilitate epistemic exclusion allows us to be attentive to forms of injustice that can arise. For example, it encourages us to consider how particular practices and cultural norms that surround the assessment of capacity may be prone to generate epistemic injustice.^61^ We discuss three salient forms of injustice below. These examples are intended to be illustrative, rather than attending to all forms of injustices that might arise in this context.^62^

### A. Capacity, deflated credibility, and testimonial injustice

The first form of epistemic injustice we focus on is testimonial injustice, where a hearer gives deflated credibility to a speaker’s testimony as a result of prejudice. This failure to perceive the speaker as credible wrongs them in their capacity as a knower. Because a person’s credibility will also be evaluated during capacity assessments, it is important to be mindful of the ways in which conferrals of credibility are influenced by interpersonal, social, and structural forms of prejudice and bias. Importantly, judgements on credibility interact closely with relations of domination based on disability, gender, and race (amongst other characteristics, including in this context, mental illness). As Cynthia Townley argues, ‘it is commonplace that gender, race, class, age, dis/ability, cultural affiliation and so on can make enormous difference as to who is believed’.^63^ The relevance of credibility in this context means there is a potential for these pre-existing biases to influence the assessment process inadvertently. Testimonial injustice may therefore arise where marginalized social groups are subject to capacity assessments, and where prejudice means they are considered less credible by the assessor when attempting to account for decisions they wish to make, leading to constraints on their epistemic agency.

This is a particularly pertinent concern given that, as noted above, capacity law applies almost exclusively to disabled people. As various theorists note, mentally disabled people and those diagnosed with mental illnesses are routinely considered to have impaired credibility due to stereotypes that they are emotionally unstable, untrustworthy, or lacking capacity for rational thought, and often denied status as epistemic agents as a result.^64^ Such attitudes can also be compounded at the intersection of disability with characteristics such as race and gender, which can make a person less likely to be trusted and believed. Whilst capacity assessments purport to be as neutral as possible and to avoid such biases (eg the MCA makes clear that a lack of capacity cannot be established merely by reference to a person’s condition, age, or appearance),^65^ as Kong argues, ‘hidden prejudices, or rigid certainty about them, can unduly orientate capacity adjudications’.^66^ A risk remains that inbuilt social attitudes and prejudices which surround disability, race, gender, and so forth may lead to certain persons being more readily denied credibility, and therefore may be less likely to be considered capacitous.

As an example of the kinds of biases or prejudices that may impact the assessment of capacity, consider the consent to sex case law. Following the Supreme Court judgment *A Local Authority v JB*,^67^ several cases have focused on the capacity of men with autistic spectrum disorders and learning disabilities to engage in sexual relations.^68^ As various commentators have noted, persons with these diagnoses are subject to prejudicial attitudes that portray them as sexually problematic and risky.^69^ In such cases, these attitudes may influence conferrals of credibility when capacity is assessed. In *Re ZZ*, for example, ‘Peter’, who was diagnosed with Mild Learning Disability and ADHD, was said to be clear in his capacity assessment that his girlfriend, Jenny, ‘would have the right say no, and he would respect that’.^70^ However, the psychiatrist assessing him considered that he lacked capacity because his impulsivity would leave him unable to control his sexual urges, and that the risk of offending behaviour was significant.^71^ Here, his words (that he would respect the other person’s refusal) were not accorded credibility. Whilst such cases are inherently challenging, it is notable here that the focus on the risk of what Peter could potentially do, in line with these dominant ideas about the sexuality of autistic men, overrode the testimony he gave.

As Scully argues, instances of testimonial injustice are not only harmful to persons within this group by failing to treat them as epistemic agents (and thereby denying them equal moral status); they can also impact their ability to trust in their own ‘narratives and judgments and, if internalized, their confidence in their overall agential capacity’.^72^ These risks, she suggests, are particularly profound for disabled people, who regularly have their status as persons of equal moral value questioned.^73^ Here, failing to accord credibility to persons subject to capacity assessments may have the effect of undermining their own perception of credibility, and potentially make it harder in the future for them to persuade others of their epistemic agency.

Where the credibility of particular groups is systematically undervalued, this can help to produce and sustain what Dotson terms ‘epistemically disadvantaged identities’, where persons who hold marginalized social identities—particularly at the intersection of characteristics such as race, gender, and crucially in this context, disability or mental illness—are less likely to be seen as credible knowers and are routinely disbelieved and silenced.^74^ When considering structural patterns that exist in assessments of capacity, such injustices can arise. Take, for example, case law in the Court of Protection concerning childbirth. On numerous occasions, the Court has held that the woman at the centre of the case either lacks capacity to make decisions about her obstetric care, or is likely to lose capacity in the future in this regard.^75^ These decisions take place in a social context where pregnant and birthing people are often considered overly emotional and unreliable, and are also expected to prioritize the well-being of the foetus and therefore accept medical advice and intervention.^76^ The women in these cases are not judged to be credible decision-makers around labour, often because they are said to lack an understanding of labour or the associated risks, including those which would put the foetus in danger. For example, in *Guys and St Thomas NHSFT v X*, the woman (who was refusing intervention) was said to lack capacity to consent to a caesarean section because she was be ‘unable to reconcile her conflicting beliefs’ between ‘wanting a natural birth and wanting a live, well, and safely born baby’ and therefore could not weigh the pros and cons of intervention.^77^ In *Re CA*, the woman expressed a strong desire to deliver vaginally, in part because of a fear of hospitals (which were stated to relate to FGM and abdominal cutting that took place in her childhood).^78^ However, she was judged—as a result of autism—to have little understanding of ‘the realities of childbirth’ and was said to be ‘very selective in retaining the information she wanted to retain, dismissing other information she did not want to hear.’^79^ Note in both these cases that the ways in which the speaker is able to project her authority and her understanding of the risks are measured against existing and accepted notions of how she ought to behave, and the logical links she ought to express to appear credible in her choices. For X, the holding of two apparently conflicting views (her desire for the more ‘unsafe’ option and her wish for a healthy child) meant she was not considered credible in her refusal of intervention. For CA, her rejection of certain aspects of medical advice and refusal to understand ‘reality’ meant she was also not considered credible in her desire for vaginal birth, despite there possibly being very compelling reasons for this (eg her previous experiences). This is not to say that such concerns were unfounded, but rather to highlight how these cultural notions of what behaviour is acceptable can influence conferrals of credibility.

Placing these cases within the broader context, it is notable that in many other childbirth cases, the woman’s lack of compliance, behavioural problems, or lack of understanding of labour and the risks to her foetus and herself are emphasized when the court finds she lacks capacity, with comparatively little emphasis placed on her testimony or views (which are at times entirely absent).^80^ Whilst none of the cases are decided by reference to the woman’s gender or disability, a notable pattern is created here that pregnant women with mental disabilities or illness are not necessarily to be considered credible in the choices they attempt to make around childbirth. As Medina argues, presumptions of epistemic authority and credibility are often based on social disparities^81^—here, there appears to be a presumption that disabled women making decisions about childbirth are not necessarily credible or to be trusted to make the ‘right’ choice. In other words, an assessment that is supposedly based on pure process is also affected by a substantive expectation of a correct choice and the perception of the social position of the speaker (ie her position as a disabled/mentally ill woman). This systematic interpretation of credibility acts to reify both patriarchal ideas of female unreliability (particularly surrounding birth),^82^ as well as prejudicial stereotypes regarding the unreliability of those with mental health disorders and disabilities, thus further epistemically disadvantaging women with similar intersectional identities.

A similar example can be found in the anorexia case law. As numerous commentators have noted, there has never been a case where a woman with anorexia has been found to have the capacity to make decisions regarding nutrition and hydration; there appears to be a presumption of *in*capacity operating in such cases.^83^ As one of us has highlighted elsewhere, despite the women in these cases making significant efforts to demonstrate they had weighed and considered their decision carefully (including for example going along with a treatment regime which was distressing in order to prove they had capacity to later refuse),^84^ prevailing ideas about the overwhelming nature of anorexia instead tend to dominate, and are used to find the women lack capacity.^85^ We do not seek to deny that anorexia impacts a person’s cognition in relation to food; we would, however, argue that the patterns created by these cases help reinforce the idea that a person with anorexia will *always* be unreliable when it comes to such decisions, and should *never* be afforded credibility. It is the pattern of unreliability created by these cases, which means that women with anorexia may be epistemically disadvantaged.

Furthermore, the structures surrounding the assessment of capacity can also increase the risk of testimonial injustice. As we noted above, the structures of modern healthcare render ill persons particularly vulnerable to epistemic injustice because certain ways of articulating and presenting knowledge are privileged, making it harder for some individuals to make themselves intelligible to (and be recognized as epistemic agents by) professionals.^86^ Such issues can be exacerbated in the context of mental disability and mental illness, where patients are subject to stigma and negative stereotypes, and may be more likely to be considered an ‘object of inquiry’ rather than a participant in the clinical encounter.^87^ These critiques not only highlight an inherent imbalance in epistemic power between the patient and professional, but also emphasize that the assessment of a person’s testimony is subject to structural pressures of the healthcare system, including ‘time pressures, routinisation of tasks, formalisation of diagnostic practices’ and so forth.^88^

These structural issues are particularly relevant when it comes to assessing capacity—which is arguably one such routine (albeit not always well-understood)^89^ healthcare practice. As argued above, being found to have capacity in part relies on a person’s ability to render themselves intelligible to the assessor; to share their knowledge and account in a manner which the assessor can rationalize through the functional test (ie demonstrative of an ability to understand/use/retain and weigh information). And, as we noted above, it remains for the decision-maker to determine what the ‘matter’ requiring a decision is, and what information is considered relevant to that decision. This may mean that other aspects which the person considers important can be rendered irrelevant to the question of their capacity.

For instance, in *North Bristol NHS Trust v R*, the ‘matter’ requiring a decision was held to be ‘whether or not her baby should be delivered pre-term by means of an elective Caesarean section’,^90^ as opposed to making decisions around different options for childbirth. This meant that the information relevant to the decision—and what formed the basis of the capacity assessment—focused on the medical risks, benefits, and practicalities of a caesarean, rather than other options around birth.^91^ Whilst it is unclear from the case what R’s desires were regarding the birth of her child, there is a notable absence of input from her on how this decision is cast and how the question of her capacity is approached.

These two aspects suggest a particularly profound imbalance in epistemic power, where the assessor determines the focus of the exchange and the key topics of relevance. Their epistemic authority and credibility in this respect are not open to question, contrasting with the open scrutiny of the person’s credibility. A tendency to favour certain kinds of expression or articulation raises the risk that particular kinds of testimony—and those coming from marginalized epistemic agents—can be subject to heightened scrutiny, leading to the deflated credibility of that group. In other words, the testimonial context here is heavily subject to the constraints created by institutional structures and further influenced by the broader structural inequalities that align with marginalized identities, creating particularly fertile ground for testimonial injustice to arise.

### B. What’s the harm? Best interests and the derivatized epistemic subject

Aside from what occurs during the assessment of capacity, it is also important to consider epistemic injustices that may arise through the assessment of best interests, which flows from a finding that a person lacks capacity. As we argued above, being found to lack capacity sees a person epistemically excluded from further decision-making. Importantly, when the decision is made through assessment of their best interests, the person’s knowledge and word alone is not relied upon to determine what should happen, but rather the decision-maker must account for ‘all the relevant circumstances’ to determine what course of action would be in their best interests, including but not limited to consideration of their wishes and feelings on the subject.^92^ The best interests test aims to navigate the balance between protecting the person, ensuring their views are accounted for, and finding practical solutions to challenging situations and problems.^93^ However, it also places an inherent limitation on the person’s epistemic agency—their interaction with others in the epistemic community is much more constrained, with their participation in discussions limited to (and understood via) the process of assessing their best interests. Again, however, there is a need to consider where this exclusion gives rise to epistemic injustice.

Fricker suggests that the primary wrong of epistemic injustice is epistemic objectification, where the hearer does not perceive the speaker as an epistemic subject—a holder/giver of knowledge who can participate in testimonial exchange—but rather an object, a mere ‘source of information’ which another can take knowledge from.^94^ Injustices of these kinds can, we would suggest, occur via the assessment of best interests; for example, if a person’s views on the potential decision are not sought, if they do not actively participate in the process, or their opinions are given too little weight due to being accorded deflated credibility. For example, Lindsey has argued that in the Court of Protection, the absence of the subject from proceedings is a form of testimonial injustice, in that paternalistic attitudes that they are too vulnerable to give evidence leads to their exclusion from proceedings, failing to value them as a giver of knowledge.^95^ Similarly, various theorists have pointed to the notable absence of the voice of the woman in the childbirth cases, and have argued that her wishes and feelings are often given little weight when her best interests are assessed.^96^ This is suggestive of these women being treated as sources of information, rather than as informants capable of influencing the best interests determination.

Yet it is important to note that the MCA emphasizes that the person *should* (at least in theory) participate as far as possible in the best interests process.^97^ Case law also emphasizes the importance of seeing things from the person’s perspective and standing in their shoes to consider what they would want.^98^ The test aims to ensure that their wishes are always considered, even though they have been deemed to lack capacity. There have also been notable cases where the person’s own views and perspective were the foundation of the best interests decision, and where real efforts were made to centre their voice. For example, in *Wye Valley NHS Trust v Mr B*,^99^ Mr B’s wishes and feelings were central to the best interests determination that his leg should not be amputated against his wishes. Jackson J emphasized that ‘the wishes and feelings, beliefs and values of people with a mental disability are as important to them as they are to anyone else, and may even be more important. It would therefore be wrong in principle to apply any automatic discount to their point of view’.^100^ There was a significant emphasis placed on Mr B’s personal identity when considering his wishes and feelings, and commentators have argued that this reflects a broader willingness to take a more patient-centred approach to best interests.^101^ This is not suggestive of treating the person entirely as an object; indeed, it could be argued that the best interests process can be used to help them retain status as an active participant within epistemic exchange and realize epistemic agency in spite of loss of capacity.

However, we would argue that it is important not to construe testimonial injustice too narrowly by seeing epistemic objectification as the key (or only) wrong. Pohlhaus Jr suggests that the primary wrong in cases of testimonial injustice is not that the victim is treated like an object, but instead they are treated as a derivatized subject; whilst the hearer recognizes the speaker’s ability to have experiences or desires, they only recognize elements which accord with their own views and understanding of the world. ^102^ As she explains:

we might say that the derivatizer treats the derivatized as though she has nothing unique to contribute to the intersubjective relations that maintain epistemic practices, even while he does recognize her as capable of some sorts of epistemic labor. In other words, she is treated as if her own lived experience from which she draws in order to add to the communal knowledge pool is simply a mirror (or perhaps shadow) of his own, but certainly not capable of contributing to our understanding of the world beyond (and in ways that might change the shape of) the scope of the derivatizer’s experienced world.^103^

This, we would argue, is a better way to characterize the epistemic wrongs that can be produced in best interests assessments. Even where there is an effort to identify the person’s wishes and feelings and encourage their participation, the requirement under the MCA is to ‘consider’ the person’s wishes and feelings.^104^ This requirement does not mean that the person’s wishes and values as a full epistemic subject must be *respected or taken seriously*. Indeed, as Series notes, ‘the structure of the best interests checklist under the MCA affords decision makers considerable discretion in how much weight they place on a person’s wishes and feelings’.^105^ The assessor retains power over the weight given to the person’s viewpoint as compared to other forms of knowledge, such as medical and clinical viewpoints. Further, the assessor retains the ability to disregard views expressed by the person that they consider to be irrelevant or potentially unwise. Once again, judgements on credibility become important—if a person’s wishes and feelings are not perceived as credible, then they may be more readily discounted. And, similarly to our discussion above, whether the person’s views *are* considered credible will again turn on whether the assessor recognizes them as appropriate or relevant. As with determining ‘the matter’, the epistemic power lies with the assessor, who undertakes a filtering process of the person’s wishes and values. This does not necessarily treat them as a full epistemic subject—it instead speaks to a truncated form of subjectivity where certain aspects of testimony can be disregarded.

Returning to the childbirth cases for an example, when considering the woman’s wishes and feelings as part of best interests, in many cases judges have inferred that they would (most likely) desire a healthy baby,^106^ including in certain cases where the woman has not given testimony on what she would want.^107^ In *North Bristol NHS Trust v R*, the judge gave the following summary of R’s wishes:

[…]whilst in her conversation with the representative of the Official Solicitor, R did at one point state when asked about the baby “no, even if it is dead it has nothing to do with me”, as I have recounted there is also evidence that R wishes to give birth to a live, healthy child, inferred from her showing occasional interest in the baby, such as asking for scan photos and wanting baby clothes, and speaking about going to see the baby. The same may be inferred from the fact that, whilst I am satisfied that she does not have capacity to consent, R has not objected to date to an elective Caesarean.^108^

And in *GSTT and SLAM v R*, R was said to have told staff that a caesarean section would be ‘the last thing she would want’.^109^ Hayden J was notably careful to consider this as a refusal, rather than as R suggesting she would consent as a last resort, and went on to note:

In this case it may be that R’s instincts and intuitive understanding of her own body (which it must be emphasised were entirely correct) led to her strenuous insistence on a natural birth. Notwithstanding the paucity of information available, I note that there is nothing at all to suggest that R was motivated by anything other than an honest belief that this was best for both her and her baby […] In the circumstances therefore, it seems reasonable to conclude that R would wish for a safe birth and a healthy baby.^110^

In these two cases, the discussion shows an acknowledgement that the woman *are* capable of some form of epistemic agency, in that even if they lack capacity, there is a recognition that they can hold their own views about the birth and that these are relevant. However, it is notable that the views they hold are represented only in a particular and narrow manner, drawing on gendered expectations (noted above) that women ought to desire above all deliveries which are safest for the foetus,^111^ and that they would opt for these if they could.^112^ This is in part a requirement of the best interests process in the court setting; judges must weigh and consider what the person has said, and represent this as part of their final determination, so to some extent cannot escape the possibility of derivitization. However, it also means that certain elements of testimony are emphasized to a much greater extent—in these two cases, the views emphasized are those which support a safe birth. And, because a desire for the safest delivery is inferred in cases where there is no testimony from the woman, this appears to suggest that the only credible view in this context is to prioritize the foetus. This again creates a pattern that a person will only be treated as an epistemic agent where their views accord with broader dominant forms of knowledge—in this instance, gendered and patriarchal perceptions of what constitutes ‘good’ motherly behaviour.^113^ This fails to treat the women as complete epistemic subjects.

What about in cases where the person’s expressed wishes and feelings are central to the determination? We would suggest that there is a need to consider the structures and circumstances surrounding the decision, which have a bearing on what views are valued as credible and relevant. It bears remembering that the best interests test operates in the context where the medical perspective is considered authoritative and ‘effectively constitutes a monopoly on the way the experience is interpreted’.^114^ As one of us has argued elsewhere, cases where best interest decisions reflect the person’s wishes and feelings are often also those where those wishes align with clinical views on the matter.^115^ The recognition of the person as credible appears to hinge on whether the content of their testimony accords with dominant medical ways to understand the world, again suggesting derivitization.

Moreover, testimonial injustice may also arise where anything that does not accord with the decision-maker’s subjectivity is left unrecognized within the assessment process. The anorexia case law is a useful illustration of this concern. Recent cases have generally held that it is not in the best interests of the woman in question to be subjected to forced treatment against her will.^116^ However, as Clough argues, these determinations are ‘not necessarily a true reflection of the complexity of their views’.^117^ Discussing the cases of *Re L*^118^ and *Ms X*,^119^ she notes that, as well as stating they did not wish to be force-fed, both women also expressed a wish not to die, with L in particular expressing a desire to be treated in a nursing home.^120^ Whilst emphasis in these cases was placed on supporting the women’s desire not to be force-fed (which was also the focus of medical evidence), there was relatively little engagement with these alternative desires when it came to best interests.^121^ The failure to focus closely on these wider views again suggests a derivitization, in that only one aspect of these women’s wishes—those which accord with the broader medical consensus (that force feeding should not happen)—was emphasized. Whilst this is not stated explicitly in these cases, we would argue that the alternative wishes the women expressed were not given due consideration as credible wishes. Indeed, as the judge emphasized in relation to *Ms X*, she would almost certainly die without the intake of nutrition.^122^ Yet it is arguably possible to develop and rely on epistemic tools that draw attention to the wish ‘not to die’ as the credible wish without requiring the established underlying logic between intake of food and survival. However, the best interests approach makes this difficult to do because the assessor is required to filter these wishes for relevance. Rather than considering and accounting for the testimony of a person in all its complexity, the decision-maker can recognize only specific elements of testimony.

### C. Narrow epistemic resources

Moving from concerns of testimonial injustice, we would also argue it is important to be mindful of forms of hermeneutical injustice that can arise when it comes to assessing capacity and best interests. As noted above, this form of injustice speaks to prejudice within epistemic resources that are utilized as part of testimonial exchanges, and how this results in unjust infringements on epistemic agency. This includes, importantly, the epistemic tools that are used to confer credibility. Here, we would emphasize that the epistemic resources that are used to assess capacity and best interests (and therefore to curate credibility) are inherently narrow. Crucially, the test for capacity relies on a medical model of disability; as noted above, the person’s inability to make a decision must be caused by the impairment of the mind or brain.^123^ As Clough argues, this ‘perceived necessity of the diagnostic element’ means that mental capacity is ‘seen as a medical concern, firmly within the realms of medical expertise’.^124^ We have similarly alluded above to the significant weight given to medical opinion and expertise when it comes to determining capacity and best interests. This suggests a heavy reliance on scientific and medical knowledge in this context, rather than on the experiential perspective of disabled people or those with mental illness. As various theorists recognize, such reliance can be exclusionary to other forms of knowing.^125^ For example, disability scholars have criticized the medical model of disability for perpetuating ableist perceptions of disability as defective and pathological.^126^ Wieseler argues that this dominant conception of disability creates significant obstacles to disabled persons’ participation in epistemic communities (and as such infringes on their epistemic agency):‘[k]nowledge and skills grounded in disabled people’s experiences are treated as unintelligible within an ableist hermeneutic’.^127^ Scrutton further suggests that the reliance on medical knowledge in the context of mental illness means that healthcare professionals essentially hold a monopoly over interpreting the person’s word: ‘the ability to interpret the experience correctly is perceived as lying with the physician, the health expert’.^128^ She argues that this hermeneutically marginalizes the person—their perspective on their experience, whilst valid, is lost because of the dominance of an also valid medical perspective.^129^

We do not aim to suggest that medical knowledge is irrelevant when it comes to assessing capacity—rather, we call attention to how an overreliance on medical epistemic resources can lead to hermeneutical and contributory injustice. Concerning the former, a medicalized approach to assessing capacity may be inapt to capture the range of experiences of disabled people, hindering their ability to communicate such experiences effectively. The focus of capacity (and credibility) on the possession of characteristics such as rationality, ability to reflect, to listen to others, etc will always be exclusionary to persons who use epistemic tools differently, making it harder for them to make themselves intelligible to the assessor, or participate meaningfully in processes. As Dotson argues, ‘being unable to communicate large portions of one’s experiences due to the deficient nature of dominant, shared epistemic resources […] profoundly impacts one’s ability to contribute to knowledge production’, because it prevents the person’s ability to render their experience intelligible.^130^

This puts those who are being assessed in particular positions of disadvantage. The epistemic subject is simply not bringing to the table those hermeneutic tools that are routinely recognized and understood by assessors. Equally, the tools made available to assessors render them unable to include and translate such information and experiences that may be relevant to their evaluation. The resulting impoverishment of our common hermeneutical resources makes it even more challenging to meaningfully recognize an agent’s epistemic authority when it comes to their care. Furthermore, such shortcomings in our epistemic resources might be further exacerbated by inattention to gendered, racial, class-based, and other social inequalities that are built into resources developed for and by dominant groups.

## V. IMPLICATIONS

As we have argued above, recognizing that the assessment of capacity also involves judgements on credibility can highlight the ways in which this process may be prone to producing forms of epistemic injustice. Such a lens has much to offer for capacity scholarship. Importantly, it draws our attention to questions beyond whether and how a person’s autonomy can be respected in capacity and best interests—something which is often a focus of debate in this area.^131^ By concentrating on unwarranted denials of epistemic agency, as opposed to autonomy, our attention is directed to wrongs that preclude a person from being able to engage with their epistemic communities as a knower; aspects that prevent them from being trusted and acknowledged by others.^132^ This has important implications both for a person’s moral status and their social status, offering a broader perspective than a focus on autonomy. An epistemic injustice lens also places crucial focus on the role of social identities and power relations of gender, disability, race, class, and so forth in determining who is believed and what knowledges are recognized and valued. This has the potential to help capacity scholarship be more mindful of how the legislation and its application interface with these wider structures of inequality. Moreover, this lens can also provide new insights into the kinds of reform that may be required to prevent or mitigate such injustices. Dotson provides a useful way to consider this. She uses a ‘degrees of change’ lens to point to the three kinds of reforms that would be required, at a minimum, to address different types of epistemic injustice.^133^

Beginning with testimonial injustice, Dotson suggests that, to address this, we need to improve how credibility is conferred to avoid unjust devaluations of credibility and the creation of epistemically disadvantaged identities.^134^ Here, the focus is placed on ensuring that the concepts we use as part of assessing credibility are operating properly. For capacity law, we would argue that this entails reflecting on how capacity and best interests are assessed under the present legal framework. Indeed, commentators have suggested several ways in which the MCA could be better applied. For example, Kong highlights the need for a relational approach to capacity.^135^ Ruck Keene similarly argues that assessors should be more alive to their own positionality and the values they bring to assessing capacity.^136^ An epistemic injustice lens could build on such approaches, in that it helps to call attention to credibility-related biases and how these can influence capacity and best interests. Additionally, the insights noted above highlight a need to reflect on imbalances of epistemic power within the assessment process, for example, concerning who identifies and conceptualizes ‘the matter’ in question, as well as how a person’s wishes and feelings are positioned. Crucially, to avoid derivatization in best interests, there is a need to reflect on the role of the person in the two core assessments, and how these can be approached to ensure that the full subjectivity of a person is recognized, rather than only elements which the decision-maker considers pertinent or relevant.

Secondly, as Dotson notes, addressing inefficiencies within our system will not necessarily be sufficient to tackle epistemic injustice; it is not enough to make our concepts work better for us if these concepts are insufficient when accounting for particular kinds of experiences.^137^ This requires second-order change—turning our attention to the epistemic resources themselves and considering whether the concepts we use *are* appropriate.^138^ As Scrutton argues, a key problem in psychiatric diagnosis is that ‘experiences reported can be forced into an existing [mould] to the exclusion of other aspects of experience that are important to the patient’.^139^ She suggests that

recognising the ways in which people diagnosed with mental illness have access to distinctive and/or unique forms of knowledge can correct our testimonial sensibilities and provide us with new hermeneutical resources.^140^

We would argue that similar reflection is needed in this context. Where forms of hermeneutical injustice are produced through capacity’s narrow hermeneutical resources, we need to consider whether the concepts themselves require reform to be more responsive to other experiences. Rather than reforming how capacity and best interests assessments are undertaken, a second-order change would involve considering how these concepts could be revised. Suggestions for conceptual reform in existing literature include untethering the assessment of capacity from diagnosis^141^ or incorporating insights from the CRPD, such as supported decision-making, into legal frameworks.^142^ A lens of epistemic injustice can help assess the desirability of these reforms by encouraging us to consider whether these new or amended concepts do sufficiently recognize different forms of knowledge which have been excluded from the ‘collective stock’,^143^ or can offer ways to tackle structural inequalities concerning credibility.

Finally (and most challengingly), Dotson suggests that we ought also to attend to the ways in which our epistemic resources are not only insufficient but *inadequate—*that is where they are simply incapable of accounting for particular kinds of insight.^144^ This can lead to cases of contributory injustice; dominant groups may refuse to engage with the epistemic resources developed by marginalized groups if they are unable to recognize or suspect their own epistemic limitations.^145^ This third order of change requires us to reflect on our shared social imaginaries and the limits of our extant epistemic systems; it requires a recognition that something valuable is currently missing if we do not step outside of our current system. In the capacity context, it means considering whether the concepts of capacity and best interests ought to be used at all or entirely replaced with alternative approaches. Indeed, the Committee on the Rights of Persons with Disabilities has directly called for the abolition of legal frameworks which deny legal capacity based on disability, and which use substitute decision-making regimes like best interests, suggesting instead that the decision made should always respect the person’s ‘will and preferences’.^146^ This call has been the subject of significant debate and critical attention in academic literature and has led to questions on whether a shift of this nature is necessary.^147^ An epistemic injustice lens again has much to offer to these debates because of its focus on a broader conception of agency, rather than the traditional approach to autonomy. For example, academic commentary on the will and preference paradigm has questioned how apparently conflicting beliefs could be reconciled.^148^ We would suggest that debates surrounding this ought to consider the role conferrals of credibility may play when identifying a person’s will and preferences, as well as how such tools can adequately serve alternative ways of knowing (eg that do not discount views as lacking credibility because they conflict) and ensure a person’s experience or knowledge is not narrowly construed.

Importantly—and as Dotson emphasizes—these orders of change are not necessarily discrete, and consideration of all three together may be required to properly tackle epistemic injustices.^149^ For example, undertaking capacity assessments *better* without changing the epistemic resources they rely upon leaves hermeneutical injustices untouched. Similarly, failing to engage with or discounting alternative visions, and simply reinforcing our present concepts whilst knowing they may be inapt can give rise to contributory injustice. This suggests a need to consider carefully what capacity law may be precluding us from understanding and knowing, as well as what kinds of knowledge we are currently missing out if we *only* focus on reforming the concepts we already have.^150^

## VI. CONCLUSION

There is much to be gained by considering the operation of mental capacity law (and the MCA in particular) from an epistemic perspective. Recognizing that judgements are made on a speaker’s credibility when assessing their capacity allows us to be mindful of the ways in which the legislation can operate to produce different forms of epistemic injustice. We focused on three key examples. First, we highlighted the ways in which structural and interpersonal biases can influence conferrals of credibility when a person’s capacity is being determined. As a form of testimonial injustice, this deflation of credibility goes beyond those often highlighted in the healthcare scholarship and is perniciously situated at the intersection of decision-making, disability, and other social inequalities, such as those related to gender. Secondly, we argued that the best interests process may be liable to treat the person as a derivatized subject and can fail to account for the full remit of their subjectivity. While current processes (including functional tests and best-interest considerations) are aimed at protecting subjective preference, we argued that there remains a significant risk that a person’s agency is only recognized as valid and credible insofar as the content of their view falls within accepted standards of medical and legal positions. Third, we suggested that the narrowness of epistemic resources that are used in the assessment of capacity may lead to the exclusion of particular forms of knowledge and may risk missing something that could be valuable when these decisions are made. It is not merely that existing tools and skills require refinement or additional consideration. Instead, an approach is required that re-examines current concepts such as capacity (and autonomy) and its recognized parameters. A focus on the epistemic nature of capacity assessments, and the wrongs that may arise in this context might highlight the legal and ethical limitations of current procedures. These examples are not intended to be exhaustive. They are rather intended to illustrate how further attention to the relationship between capacity assessments and credibility can highlight specifically epistemic wrongs. Reflecting on this, we have suggested insights that an epistemic lens can bring to questions of reform and pointed to different kinds of change that would need to be considered as part of tackling or mitigating forms of epistemic injustice, and recognizing and respecting patients as contributing members to the epistemic communities and systems involved in their care.

## Footnotes

1 See eg Havi Carel and Ian James Kidd, ‘Epistemic Injustice in Healthcare: A Philosophial Analysis’ (2014) 17 Medicine, Health Care, and Philosophy 529; Jackie Leach Scully, ‘From “She Would Say That, Wouldn’t She?” To “Does She Take Sugar?” Epistemic Injustice and Disability’ (2018) 11 International Journal of Feminist Approaches to Bioethics 106; Christine Wieseler, ‘Epistemic Oppression and Ableism in Bioethics’ (2020) 35 Hypatia 714.

2 Gaile Pohlhaus Jr, ‘Varieties of Epistemic Injustice’ in Ian James Kidd, José Medina and Gaile Pohlhaus Jr (eds), *The Routledge Handbook of Epistemic Injustice* (Routledge, Taylor & Francis Group 2017) 13.

3 Kristie Dotson, ‘Conceptualizing Epistemic Oppression’ (2014) 28 Social Epistemology 115, 115.

4 Kristie Dotson, ‘A Cautionary Tale: On Limiting Epistemic Oppression’ (2012) 33 Frontiers (Boulder) 24; Kristie Dotson, ‘Tracking Epistemic Violence, Tracking Practices of Silencing’ (2011) 26 Hypatia 236; Miranda Fricker, *Epistemic Injustice: Power and the Ethics of Knowing* (OUP 2007).

5 For example, Paul Crichton, Havi Carel and Ian James Kidd, ‘Epistemic Injustice in Psychiatry’ (2017) 41 BJPsych Bulletin 65; Josh Dohmen, ‘Interactions with Delusional Others: Reflections on Epistemic Failures and Virtues’ in Adam Cureton and David T Wasserman (eds), *The Oxford Handbook of Philosophy and Disability* (OUP 2020); Natalie Dorfman and Joel Michael Reynolds, ‘The New Hysteria: Borderline Personality Disorder and Epistemic Injustice’ (2023) 16 International Journal of Feminist Approaches to Bioethics 162; Lisa Bortolotti (ed), *Epistemic Justice in Mental Healthcare: Recognising Agency and Promoting Virtues Across the Life Span* (Springer Nature 2025) <https://library.oapen.org/handle/20.500.12657/96133> accessed 24 August 2025.

6 See eg Jaime Lindsey, ‘Testimonial Injustice and Vulnerability: A Qualitative Analysis of Participation in the Court of Protection’ (2019) 28 Social & Legal Studies 450; Camillia Kong, *Mental Capacity in Relationship: Decision-Making, Dialogue, and Autonomy* (CUP 2017) 215–224.

7 Piers Gooding, ‘Supported Decision-Making: A Rights-Based Disability Concept and Its Implications for Mental Health Law’ (2013) 20 Psychiatry, Psychology and Law 431; Alex Ruck Keene and others, ‘Mental Capacity—Why Look for a Paradigm Shift?’ (2023) 31 Medical Law Review 340; Peter Bartlett, ‘Re-Thinking the Mental Capacity Act 2005: Towards the Next Generation of Law’ (2022) 86 Modern Law Review 659.

8 Ian James Kidd, Jose Medina and Gaile Pohlhaus Jr (eds), *The Routledge Handbook of Epistemic Injustice* (Routledge, Taylor & Francis Group 2017) 1.

9 For a detailed exploration of different forms of epistemic injustice and particular contexts these arise, see Kidd, Medina and Pohlhaus Jr (n 8).

10 Fricker (n 4).

11 Patricia Hill Collins, *Black Feminist Thought: Knowledge, Consciousness, and the Politics of Empowerment* (30th Anniversary edition, Routledge 2022); Charles W Mills, *The Racial Contract* (Cornell University Press 1999); Andrea J Pitts, ‘Decolonial Praxis and Epistemic Injustice’ (n 2).

12 Pohlhaus Jr (n 2) 14–15.

13 Cynthia Townley, ‘Trust and the Curse of Cassandra (An Exploration of the Value of Trust)’ (2003) 10 Philosophy in the Contemporary World 105, 107–108.

14 Jackie Leach Scully, ‘Epistemic Exclusion, Injustice, and Disability’ in Adam Cureton and David T Wasserman (eds), *The Oxford Handbook of Philosophy and Disability* (OUP2020) 300; Dotson, ‘Conceptualising Epistemic Oppression’ (n 3) 115.

15 Scully (n 14) 300.

16 ibid.

17 Dotson (n 3) 115; Scully (n 14) 300.

18 Fricker (n 4) 1.

19 Townley (n 13) 106.

20 ibid 108.

21 José Medina, ‘The Relevance of Credibility Excess in a Proportional View of Epistemic Injustice: Differential Epistemic Authority and the Social Imaginary’ (2011) 25 Social Epistemology 15, 18.

22 Townley (n 13) 106.

23 Medina (n 21) 20.

24 Dotson, ‘A Cautionary Tale’ (n 4) 29; Fricker (n 4) 155.

25 Dotson ibid; Dotson (n 3) 127.

26 Carel and Kidd (n 1) 530.

27 Dotson, ‘A Cautionary Tale’ (n 4) 31–32.

28 ibid 32.

29 Havi Carel and Gita Györffy, ‘Seen but Not Heard: Children and Epistemic Injustice’ (2014) 384 The Lancet 1256, 1257.

30 Anastasia Philippa Scrutton, ‘Epistemic Injustice and Mental Illness’ in (n 2) 347.

31 Stephanie LeBlanc and Elizabeth Anne Kinsella, ‘Toward Epistemic Justice: A Critically Reflexive Examination of “Sanism” and Implications for Knowledge Generation’ (2016) 10 Studies in Social Justice 59, 63–64.

32 Carel and Kidd (n 1) 531–532.

33 Fiona Kumari Campbell, ‘Inciting Legal Fictions: “disabilities” Date with Ontology and the Ableist Body of the Law’ (2001) 10 Griffith Law Review 42; Daniel Goldberg, ‘Epistemic Injustice, Disability Stigma, and Public Health Law’ in I Glenn Cohen and others (eds), *Disability, Health, Law, and Bioethics* (CUP 2020).

34 See eg Fricker (n 4) ch 1; Swethaa S Ballakrishnen and Sarah B Lawsky, ‘Law, Legal Socializations, and Epistemic Injustice’ (2022) 47 Law & Social Inquiry 1026; Michael Sullivan, ‘Epistemic Justice and the Law’ in (n 2). In relation to the operation of the Court of Protection, see Lindsey (n 6).

35 Giles Newton-Howes, Simon Walker and Neil John Pickering, ‘Epistemic Problems with Mental Health Legislation in the Doctor–Patient Relationship’ (2023) 49 Journal of Medical Ethics 727, 728.

36 Law Commission, *Report on Mental Incapacity* (Law Com No 231, 1995).

37 Mary Donnelly, ‘Capacity Assessment under the Mental Capacity Act 2005: Delivering on the Functional Approach?’ (2009) 29 Legal Studies 464; Ruck Keene and others (n 7).

38 MCA 2005, s 2.

39 ibid s 5.

40 Usually in the case of serious cases or disputes between parties—see Office of the Public Guardian, ‘Mental Capacity Act Code of Practice’ (*GOV.UK*, 14 October 2020) 142 <https://www.gov.uk/government/publications/mental-capacity-act-code-of-practice> accessed 12 April 2023.

41 MCA 2005, s 3(1).

42 For a critique of this approach, see Beverley Clough, *The Spaces of Mental Capacity Law: Moving Beyond Binaries* (Routledge 2021) 60–63.

43 MCA 2005, s 4.

44 Scott YH Kim and others, ‘Broad Concepts and Messy Realities: Optimising the Application of Mental Capacity Criteria’ (2022) 48 Journal of Medical Ethics 838.

45 Fricker (n 4) 18.

46
*Lancashire & South Cumbria NHS Foundation Trust and Another v AH* [2022] EWCOP 45.

47 ibid [34].

48 ibid [51].

49 Kim and others (n 44).

50 Medina (n 21) 20.

51 Kim and others (n 44).

52 Carel and Kidd (n 1) 530.

53 MCA 2005, s 3(1).

54
*A Local Authority v JB (Rev 1)* [2021] UKSC 52 [68]–[70]; *North Bristol NHS Trust v R* [2023] EWCOP 5 [57]–[58].

55 Townley (n 13) 105–106.

56 MCA 2005, s 1(5).

57 Townley (n 13) 106.

58 Newton-Howes, Walker and Pickering (n 35).

59 Scully (n 1) 107-108.

60 Fricker (n 4) ch 2.

61 Carel and Kidd (n 1) 531.

62 For example, we recognize that issues of epistemic injustice may be different in spaces such as the Court of Protection, as compared to decision-making under s 5 MCA in health and social care settings. Our focus here is on capacity assessments generally, rather than on specific issues that arise within these contexts.

63 Townley (n 13) 109.

64 LeBlanc and Kinsella (n 31) 64; Scrutton (n 30) 348; Josh Dohmen, ‘“A Little of Her Language”: Epistemic Injustice and Mental Disability’ (2016) 93 Res Philosophica 669.

65 MCA 2005, s 2(3).

66 Kong (n 6) 217.

67
*Re JB* (n 54).

68 See eg *Re PN* [2023] EWCOP 44; *Local Authority A v ZZ* [2024] EWCOP 21; *A Local Authority v ZX* [2024] EWCOP 30.

69 Monique Huysamen, Marianthi Kourti and Christopher Hatton, ‘A Critical Overview of How English Health and Social Care Publications Represent Autistic Adults’ Intimate Lives’ (2023) 43(4) Critical Social Policy 626; Ruby Reed-Berendt and Beverley Clough, ‘(Un)Blurred Lines? Sex, Disability, and the Dynamic Boundaries of Mental Capacity Law’ (2024) 93 International Journal of Law and Psychiatry 101960; Anna Arstein-Kerslake, ‘Understanding Sex: The Right to Legal Capacity to Consent to Sex’ (2015) 30 Disability & Society 1459.

70
*Local Authority A v ZZ* [2023] EWCOP 61 [19].

71 ibid [25]–[26].

72 Scully (n 1) 111.

73 ibid.

74 Dotson, ‘Conceptualising Epistemic Oppression’ (n 3) 125; Dotson, ‘Tracking Epistemic Violence’ (n 4).

75 eg *Re JP* [2019] EWCOP 23*; North Bristol NHS Trust v R* [2023] EWCOP 5*; University Hospitals Dorset NHS Foundation Tryst v Miss K* [2021] EWCOP 40*; An NHS Trust v An Expectant Mother* [2021] EWCOP 33; *GSTT and SLAM v R* [2020] EWCOP 4. For discussions on the topic, see Sara Fovargue, ‘*Anticipating Issues with Capacitous Pregnant Women: United Lincolnshire NHS Hospitals Trust v CD* [2019] EWCOP 24 and Guys and St Thomas’ NHS Foundation Trust (GSTT) and *South London and Maudsley NHS Foundation Trust (SLAM) v R* [2020] EWCOP 4’ (2020) 28 Medical Law Review 781; Aimee V Hulme, ‘Anticipatory Declarations in Obstetric Care: A Relational and Spatial Examination of Patient Empowerment, Institutional Impacts and Temporal Challenges’ (2024) 32 Medical Law Review 530.

76 Quill R Kukla, ‘Measuring Mothering’ (2008) 1 International Journal of Feminist Approaches to Bioethics 67, 74; Stella Villarmea, ‘When a Uterus Enters the Room, Reason Goes out the Window’ in C Pickles and J Herring (eds) *Women’s Birthing Bodies and the Law: Unauthorised Intimate Examinations, Power and Vulnerability* (Hart 2020) 69.

77 [2019] EWCOP 35 [27].

78 [2016] EWCOP 51 [33].

79 ibid [25]–[29].

80 As discussed in Samantha Halliday, ‘Pregnancy and Severe Mental Illness: Birth Choices, Best Interests and the Untapped Potential of Advance Decisions’ (2024) 24 Medical Law International 14, 22.

81 Medina (n 21) 22.

82 Sara Fovargue, ‘In Whose Best Interests?: Childbirth Choices and Other Health Decisions’ (2021) 137 Law Quarterly Review 604, 18.

83 Beverley Clough, ‘Anorexia, Capacity, and Best Interests: Developments in the Court of Protection since the Mental Capacity Act 2005’ (2016) 24 Medical Law Review 434, 438; John Coggon, ‘Anorexia Nervosa, Best Interests, and the Patient’s Human Right to “a Wholesale Overwhelming of Her Autonomy”: *A Local Authority v E* [2012] EWHC 1639 (COP) [2012] HRLR 29’ (2014) 22 Medical Law Review 119, 127; Daniel Wei L Wang, ‘Mental Capacity Act, Anorexia Nervosa and the Choice Between Life-Prolonging Treatment and Palliative Care: *A NHS Foundation Trust v Ms X*’ (2015) 78 The Modern Law Review 871, 871.

84
*A Mental Health NHST v BG and others* [2022] EWCOP 26 [34].

85 Beverley Clough and Ruby Reed-Berendt, ‘Consent and Mental Capacity Law: A Story of (Dis)Empowerment and Medical Dominance’, in Louise Austin and José Miola (eds), *The Elgar Research Handbook on Informed Consent* (Edward Elgar Publishing, forthcoming 2026).

86 Havi Carel and Ian James Kidd, ‘Epistemic Injustice in Medicine and Healthcare’ in n(2) 337.

87 Crichton, Carel and Kidd (n 5) 67–68.

88 Carel and Kidd (n 86) 339.

89 See, eg, the numerous criticisms of the Act’s misapplication in practice and a lack of understanding amongst professionals in post-legislative scrutiny; House of Lords Select Committee on the Mental Capacity Act 2005, ‘Mental Capacity Act 2005: Post-Legislative Scrutiny’ (2014) HL Paper 139 <https://publications.parliament.uk/pa/ld201314/ldselect/ldmentalcap/139/13902.htm> accessed 12 April 2023.

90
*North Bristol* (n 75) [58].

91 ibid [62].

92 MCA 2005, s 4.

93 Mary Donnelly, ‘Best Interests in the Mental Capacity Act: Time to Say Goodbye?’ (2016) 24 Medical Law Review 318; Office of the Public Guardian (n 40).

94 Fricker (n 4) 131–133.

95 Lindsey (n 6) 455.

96 Hulme (n 75) 542–543; Halliday (n 80) 18; Fovargue (n 82) 623.

97 MCA 2005, s(4).

98
*Re MN* [2015] EWCA Civ 411; *Aintree v James* [2013] UKSC 67 [18].

99 [2015] EWCOP 60.

100 ibid [11].

101 Lucy Series, ‘The Place of Wishes and Feelings in Best Interests Decisions: *Wye Valley NHS Trust v Mr B*’ (2016) 79 Modern Law Review 1101, 1106; Carolyn S Johnston, ‘Lack of Capacity Is Not an “off Switch” for Rights and Freedoms: *Wye Valley NHS Trust v Mr B* (By His Litigation Friend, the Official Solicitor) [2015] EWCOP 60’ (2017) 25 Medical Law Review 662, 668.

102 Gaile Pohlhaus, ‘Discerning the Primary Epistemic Harm in Cases of Testimonial Injustice’ (2014) 28 Social Epistemology 99, 105–106.

103 ibid 106.

104 MCA 2005, s 4(6).

105 Series (n 101) 1110.

106 See discussion in Hulme (n 75) 538–539; Halliday (n 80) 28.

107 For example where they have not participated in the case, eg *Re AA* [2012] EWCOP 4378, Proceedings Index (“it must be in the mother’s interest to have a healthy baby”); *Miss K* (n 75) [18] (“I have no reason to believe her wishes would be anything other than to have the safest birth possible”); *A London NHS Trust v KB* [2020] EWCOP 59 [24] (“I am sure that, if she had capacity, KB’s priority would be to do the best for her unborn baby”).

108
*North Bristol* (n 75) [80].

109
*GSTT and SLAM v R* (n 74) [56].

110 ibid [63].

111 Hulme (n 75) 539.

112 Tasha N Dubriwny, ‘Television News Coverage of Postpartum Disorders and the Politics of Medicalization’ (2010) 10 Feminist Media Studies 285.

113 Zaina Mahmoud and Elizabeth Chloe Romanis, ‘On Gestation and Motherhood’ (2023) 31 Medical Law Review 109; Kukla (n 77).

114 Scrutton (n 30) 349.

115 Clough and Reed-Berendt (n 85). See also Halliday (n 80) 26.

116 See eg *Northamptonshire Healthcare NHSFT v AB* [2020] EWCOP 40; *BG* (n 84).

117 Clough (n 83) 443.

118
*The NHS Trust v L* [2012] EWHC 2741 (COP).

119
*A NHS Foundation Trust v Ms X* [2014] EWCOP 35.

120 Clough (n 83) 443.

121 ibid 443.

122
*Ms X* (n 119) [34]–[36].

123 MCA 2005, s 2(1).

124 Clough (n 42) 42.

125 See eg Alison Reiheld, ‘Patient Complains of…: How Medicalization Mediates Power and Justice’ (2010) 3 International Journal of Feminist Approaches to Bioethics 72.

126 Alison Kafer, *Feminist, Queer, Crip* (Indiana University Press 2013) 5.

127 Wieseler (n 1).

128 Scrutton (n 30) 349.

129 ibid 350.

130 Dotson, ‘Conceptualising Epistemic Oppression’ (n 3) 127.

131 See eg Kong (n 6); John Coggon, ‘Mental Capacity Law, Autonomy, and Best Interests: An Argument for Conceptual and Practical Clarity in the Court of Protection’ (2016) 24 Medical Law Review 396; Mary Donnelly, *Healthcare Decision-Making and the Law: Autonomy, Capacity and the Limits of Liberalism* (CUP 2010); Anna Arstein-Kerslake, *Restoring Voice to People with Cognitive Disabilities: Realizing the Right to Equal Recognition before the Law* (UP 2017) 86–96.

132 Townley (n 13) 109.

133 Dotson, ‘Conceptualising Epistemic Oppression’ (n 3); Pohlhaus Jr (n 2) 19–20.

134 Dotson, ‘Conceptualising Epistemic Oppression’ (n 3) 125–126.

135 Kong (n 6).

136 Alex Ruck Keene, ‘Is Mental Capacity in the Eye of the Beholder?’ (2017) 11 Advances in Mental Health and Intellectual Disabilities 30.

137 Dotson, ‘Conceptualising Epistemic Oppression’ (n 3) 126–127.

138 ibid 128.

139 Scrutton (n 30) 349.

140 ibid 347.

141 See eg Eilionóir Flynn and Anna Arstein-Kerslake, ‘State Intervention in the Lives of People with Disabilities: The Case for a Disability-Neutral Framework’ (2017) 13 International Journal of Law in Context 39. Though approaches such as this are not without pitfalls—see Linda Steele, ‘Temporality, Disability and Institutional Violence: Revisiting In Re F’ (2017) 26 Griffith Law Review 378.

142 Gooding (n 7).

143 Scully (n 14) 302.

144 Dotson, ‘Conceptualising Epistemic Oppression’ (n 3) 130–131.

145 Dotson, ‘A Cautionary Tale’ (n 4) 32; Scully (n 14) 304.

146 CRPD Committee General Comment No 1.

147 Ruck Keene and others (n 7); Piers Gooding, ‘Navigating the “Flashing Amber Lights” of the Right to Legal Capacity in the United Nations Convention on the Rights of Persons with Disabilities: Responding to Major Concerns’ (2015) 15 Human Rights Law Review 45; Penelope Weller, ‘Reconsidering Legal Capacity: Radical Critiques, Governmentality and Dividing Practice’ (2014) 23 Griffith Law Review 498; Clough (n 42) 71–74.

148 Peter Bartlett, ‘Will and Preferences in the Overall CRPD Project’ (2019) 18 World Psychiatry 48; George Szmukler, ‘“Capacity”, “Best Interests”, “Will and Preferences” and the UN Convention on the Rights of Persons with Disabilities’ (2019) 18 World Psychiatry 34; Gooding (n 146).

149 Dotson, ‘Conceptualising Epistemic Oppression’ (n 3) 133.

150 Pohlhaus Jr (n 2) 18.

